# Association between herpes zoster ophthalmicus and the risk of corneal ulcer

**DOI:** 10.3389/fmed.2025.1529908

**Published:** 2025-05-12

**Authors:** Shih-Feng Weng, Yuh-Shin Chang, Jhi-Joung Wang, Han-Yi Jan, Jiun-Yi Chen, Sung-Huei Tseng, Ren-Long Jan

**Affiliations:** ^1^Department of Healthcare Administration and Medical Informatics, Kaohsiung Medical University, Kaohsiung, Taiwan; ^2^Department of Ophthalmology, Chi Mei Medical Center, Tainan, Taiwan; ^3^School of Medicine, National Sun Yat-sen University, Kaohsiung, Taiwan; ^4^Department of Medical Research, Chi Mei Medical Center, Tainan, Taiwan; ^5^School of Medicine, Tzu Chi University, Hualien, Taiwan; ^6^School of Medicine, National Yang Ming Chiao Tung University, Taipei, Taiwan; ^7^Department of Ophthalmology, National Cheng Kung University Hospital, College of Medicine, National Cheng Kung University, Tainan, Taiwan; ^8^Department of Paediatrics, Chi Mei Medical Center, Liouying, Tainan, Taiwan

**Keywords:** herpes zoster ophthalmicus, corneal ulcer, Taiwan longitudinal health insurance database, cohort study, epidemiology

## Abstract

**Introduction:**

Herpes zoster ophthalmicus (HZO) is a serious condition resulting from the reactivation of the varicella-zoster virus, affecting the ophthalmic branch of the trigeminal nerve. In HZO, exposure keratopathy can lead to a range of ocular surface disorders, including superficial punctate keratitis and disruption of the ocular surface barrier, thereby increasing the risk of infectious corneal ulcers. This study, therefore, aimed to investigate the risk of corneal ulceration in patients with HZO.

**Materials and methods:**

This nationwide, population-based, retrospective, matched-cohort study included 44,317 newly diagnosed patients with HZO, identified using the International Classification of Diseases, Ninth Revision, Clinical Modification, and selected from the Taiwan National Health Insurance Research Database. The control group, matched for age, sex, and potential comorbidities, included 132,951 patients without HZO selected from the Taiwan Longitudinal Health Insurance Database 2000. Patient data were collected between 1 January 2004 and 31 December 2011 with both groups tracked from the index date until December 2013. The incidence and risk of corneal ulcers were compared between the groups. Cox proportional hazards regression analysis was used to calculate the adjusted hazard ratio for corneal ulcer development, while the cumulative incidence rate of corneal ulcers was estimated using Kaplan–Meier analysis.

**Results:**

A total of 1,374 patients with HZO and 938 controls developed corneal ulcers during the follow-up period. The incidence rate of corneal ulcer was 4.59 times [95% confidence interval (CI) = 4.23–4.99; *p* < 0.0001] higher in patients than in controls. After adjusting for potential confounders, including diabetes mellitus, atopy trait, chronic renal disease, ocular allergic conditions, human immunodeficiency virus infection, and contact lens wearing, patients with HZO were 4.53 times more likely to develop a corneal ulcer in total cohort (adjusted HR, 4.53; 95% CI = 4.17–4.93; *p* < 0.05).

**Conclusion:**

Patients with HZO are at an increased risk of developing corneal ulcers and should be appropriately counseled regarding this risk.

## 1 Introduction

Herpes zoster ophthalmicus (HZO) is a serious condition caused by the reactivation of the varicella-zoster virus and affects the ophthalmic branch of the trigeminal nerve (1, 2). Clinical signs typically include a facial herpetic rash, often accompanied by neuropathic pain, fever, and headaches. Ocular involvement occurs in about 50% of patients, leading to inflammation of various eye structures, with potential manifestations such as conjunctivitis, keratitis, uveitis, episcleritis, scleritis, retinitis, retinal necrosis, and optic neuritis (3–6). The cornea can be affected with superficial punctate keratitis within a week, progressing to pseudodendrites. Various forms of stromal keratitis may develop 1 month to several years later, 4–6 including exposure keratopathy, caused by cicatricial changes in the eyelids that lead to corneal desiccation, and neurotrophic keratopathy, marked by reduced corneal sensation (7, 8).

Exposure keratopathy in HZO, due to cicatricial eyelid changes and inadequate tear production (7), contributes to various ocular surface disorders, including superficial punctate keratitis and compromised ocular surface barrier function, which increases susceptibility to infectious ulcers (9–11). Additionally, neurotrophic keratitis in these patients can lead to breakdown of the corneal epithelium, resulting in secondary inflammation, thinning, and heightened vulnerability to opportunistic infections (12). Furthermore, the use of topical corticosteroids and immunomodulatory eye drops commonly prescribed for HZO, as well as systemic immunosuppressive agents such as antineoplastic drugs, may contribute to the development of corneal ulcers and opportunistic infections by significantly impairing immune responses and corneal healing (13–17). Therefore, it is clinically important to investigate whether HZO serves as a risk factor for corneal ulcers.

While some studies have explored the potential link between HZO and corneal ulcers, their findings are primarily based on case reports and small case series (18, 19). To the best of our knowledge, no extensive cohort studies have explored the potential of HZO as a risk factor for the subsequent occurrence of corneal ulcers. Establishing this connection would allow for early intervention and more informed therapeutic strategies, ultimately improving patient outcomes and reducing the risk of severe vision loss. Therefore, we conducted a nationwide, population-based cohort study in Taiwan to investigate the risk of corneal ulcer development following a diagnosis of HZO.

## 2 Materials and methods

### 2.1 Database

The data for our study were sourced from the National Health Insurance Research Database (NHIRD), provided by the NHRI in Taiwan. This database includes coded information on each enrolle’s demographics, including birth date, sex, residential area, and International Classification of Diseases, Ninth Revision, Clinical Modification (ICD-9-CM) codes. It captures diagnoses, procedures, prescription details, and expenditure claims, whether the patient received inpatient or outpatient care. Because the database used in this study was de-identified, the Institutional Review Board of the Chi-Mei Medical Center in Tainan waived the need for ethical approval and informed consent.

### 2.2 Selection of patients and variables

We enrolled 44,317 newly diagnosed HZO patients (ICD-9-CM code 053.2) into our retrospective cohort study. Patient data were gathered from January 1, 2004, to December 31, 2011. We excluded patients with unspecified gender, missing demographic information, or a prior diagnosis of corneal ulcer before HZO. Corneal ulcers were identified by ICD-9-CM code 370.0, which includes conditions such as 370.00 (corneal ulcer, unspecified), 370.01 (marginal), 370.02 (ring), 370.03 (central), 370.04 (hypopyon ulcer), 370.05 (mycotic), and 370.06 (perforated), but excludes 370.07 (Mooren’s ulcer).

For each patient diagnosed with HZO, three corresponding non-HZO controls were randomly selected from the Longitudinal Health Insurance Database 2000 (LHID 2000), a subset of the NHIRD. Propensity score matching (PSM), based on age and sex, was employed using a 1:3 nearest neighbor matching algorithm without replacement to ensure comparability between groups. The NHRI employed a systematic sampling method to compile the LHID 2000, which includes comprehensive claims data for 1,000,000 beneficiaries starting from the year 2000. We matched the 132,951 non-HZO controls with the HZO patients by age (±30 days), sex, and the index date, defined as the date of the first HZO diagnosis. Controls with a prior diagnosis of HZO or corneal ulcer before the index date were excluded. This database is maintained by the Taiwan National Health Research Institutes and is accessible to the public through a formal application process.

To assess the incidence of corneal ulcers, we tracked all participants from the index date through the end of 2013 or until death, whichever occurred first, and recorded each participant’s demographic data. Additionally, we collected data on potential risk factors, including comorbidities like diabetes mellitus (20) (ICD-9-CM code 250); atopy trait [asthma (code 477), allergic rhinitis (code 493), atopic dermatitis (code 691)]; chronic renal diseases (21) (codes 585); ocular allergic conditions [allergic conjunctivitis (code 372.14), atopic keratoconjunctivitis (code 372.05), vernal keratoconjunctivitis (code 372.13, 370.32)]; human immunodeficiency virus (HIV) (22) (code 042 and V08); and contact lens wearing [corneal disorder due to contact lens (code 371.82), corneal oedema due to contact lens (code 371.24)] were collected. We only included HZO patients and their controls with these comorbidities if the condition was documented in at least three ambulatory care claims or recorded during an inpatient stay within 1 year before the index date. In addition, Supplementary Table S1 provides details on the types of corneal ulcers.

### 2.3 Statistical analysis

Statistical analyses were performed using SAS 9.4 for Windows (SAS Institute, Inc., Cary, NC, USA). Pearson’s chi-squared test was used to compare baseline demographics and comorbidities between the HZO and control groups. Corneal ulcer incidence was calculated by dividing the number of cases identified during the follow-up period by total person-years (PY) for each group, stratified by age, sex, and selected comorbidities. Poisson regression was used to obtain the incidence rate ratio (IRR), comparing the risk of corneal ulcers between the two groups. Adjusted hazard ratios (HRs) and 95% confidence intervals (CIs) for corneal ulcer risk were estimated using Cox proportional hazards regression. Kaplan–Meier curves were plotted to show cumulative incidence, with differences analyzed using the log-rank test. Statistical significance was defined as *p* < 0.05.

## 3 Results

### 3.1 Demographic data

From the beginning of 2004 to the end of 2011, after excluding ineligible subjects, 44,317 patients with HZO and 132,951 control non-HZO patients were recruited (Table 1). The average age was the same for both HZO patients and controls, 52.90 years [standard deviation (SD), 20.92 years]. Of the 44,317, 21,862 (49.33%) were men and 22,455 (50.67%) were women. The HZO group exhibited a significantly higher prevalence of comorbidities, including diabetes, atopy trait, chronic renal disease, ocular allergic conditions, and HIV infection, than the controls (*p* < 0.0001). Contact lens wearing is a risk factor for corneal ulcers but was too rare to be assessed.

**Table 1:** Comparison of the demographic characteristics and comorbidities between the herpes zoster ophthalmicus (HZO) and control groups.

Characteristics	HZO (*N* = 44,317)	Controls (*N* = 132,951)	*p*-value
Age (years; mean ± SD)	52.90 ± 20.92	52.90 ± 20.90	0.9935
	Number (%)	Number (%)	
Age group (years)			
<12	1,742 (3.93)	5,194 (3.90)	>0.9999
12–19	1,801 (4.06)	5,435 (4.09)	
20–29	4,112 (9.28)	12,336 (9.28)	
30–39	4,529 (10.22)	13,587 (10.22)	
40–49	5,613 (12.67)	16,839 (12.67)	
50–59	8,245 (18.60)	24,735 (18.60)	
60–69	7,488 (16.90)	22,464 (16.90)	
70–79	7,147 (16.13)	21,441 (16.13)	
≥80	3,640 (8.21)	10,920 (8.21)	
Sex			
Male	21,862 (49.33)	65,555 (49.31)	0.9366
Female	22,455 (50.67)	67,396 (50.69)	
Baseline comorbidity			
Diabetes mellitus	5,406 (12.20)	12,133 (9.13)	<0.0001
Atopy trait	3,143 (7.09)	5,977 (4.50)	<0.0001
Chronic renal disease	1,255 (2.83)	2,135 (1.61)	<0.0001
Ocular allergic conditions	802 (1.81)	1,165 (0.88)	<0.0001
HIV infection	79 (0.18)	26 (0.02)	<0.0001
Contact lens wearing	2 (0.00)	0 (0.00)	0.0625

Demographic characteristics and comorbidities were compared between the HZO and control groups using Pearson’s chi-square test. HIV, human immunodeficiency virus; HZO, herpes zoster ophthalmicus; SD, standard deviation.

### 3.2 Corneal ulcer incidence rates

During the follow-up period, there was a higher incidence rate of corneal ulcers in HZO patients (53.55/10,000 PY) than in controls (11.65/10,000 PY), leading to a significant difference in the IRR of corneal ulcers (4.59, 95% CI = 4.23–4.99, *p* < 0.0001) between the groups (Table 2).

**Table 2:** Risk of corneal ulcers in the herpes zoster with ophthalmic complications (HZO) and control groups.

Characteristics	HZO				Controls				IRR (95% CI)	*p*-value
	*N*	Corneal	PY	Rate^a^	*N*	Corneal	PY	Rate^a^		
All	44,317	1,374	256,596	53.55	132,951	938	804,844	11.65	4.59 (4.23–4.99)	<0.0001
Age (years)										
<12	1,742	52	13,788	37.71	5,194	52	43,939	11.83	3.19 (2.17–4.68)	<0.0001
12–19	1,801	87	11,945	72.83	5,435	114	37,043	30.78	2.37 (1.79–3.13)	<0.0001
20–29	4,112	138	26,291	52.49	12,336	152	80,720	18.83	2.79 (2.21–3.51)	<0.0001
30–39	4,529	143	27,532	51.94	13,587	99	85,023	11.64	4.46 (3.45–5.76)	<0.0001
40–49	5,613	186	33,387	55.71	16,839	101	103,081	9.80	5.69 (4.46–7.24)	<0.0001
50–59	8,245	203	46,166	43.97	24,735	136	142,848	9.52	4.62 (3.72–5.74)	<0.0001
60–69	7,488	233	42,411	54.94	22,464	125	132,682	9.42	5.83 (4.69–7.25)	<0.0001
70–79	7,147	226	38,722	58.36	21,441	129	123,285	10.46	5.58 (4.49–6.92)	<0.0001
≥80	3,640	106	16,354	64.82	10,920	30	56,222	5.34	12.15 (8.10–18.22)	<0.0001
Sex										
Male	21,862	728	123,656	58.92	65,555	423	391,127	10.81	5.44 (4.83–6.14)	<0.0001
Female	22,455	646	132,940	48.59	67,396	515	413,717	12.45	3.90 (3.48–4.38)	<0.0001
Comorbidity										
Diabetes mellitus	5,406	152	27,570	55.13	12,133	75	64,909	11.55	4.77 (3.62–6.29)	<0.0001
Atopy trait	3,143	79	17,539	45.04	5,977	41	34,881	11.75	3.83 (2.63–5.59)	<0.0001
Chronic renal disease	1,255	32	5,805	55.12	2,135	10	10,682	9.36	5.89 (2.89–11.98)	<0.0001
Ocular allergic conditions	802	36	4,132	87.12	1,165	12	6,719	17.86	4.88 (2.54–9.38)	<0.0001
HIV infection	79	3	387	77.52	26	0	134	0	0.00 (0.00–0.00)	–
Contact lens wearing	2	1	10	1,000	0	0	0	–	–	–

Poisson regression analysis was performed to calculate the incidence rate ratio. HIV, human immunodeficiency virus; HZO, herpes zoster ophthalmicus; IRR, in-cidence rate ratio; PY, person-years.

a Rate: per 10,000 person-years.

The incidence of corneal ulcers was higher in HZO patients across all age groups compared to controls, with HZO patients aged 12–19 years showing the highest incidence (72.83 per 10,000 person-years). Incidence Rate Ratios (IRR) were significantly higher across all HZO age groups compared to their age-matched control groups (Table 2). Notably, the incidence rate in HZO patients aged 80 years and older was 12.15 times that of their age-matched controls (IRR = 12.15; 95% CI = 8.10–18.22; *p* < 0.0001) (Table 2).

The incidence rate of corneal ulcers among male HZO patients was 58.92 per 10,000 person-years, compared to 10.81 per 10,000 PY for male controls (IRR = 5.44; 95% CI = 4.83–6.14; *p* < 0.0001). A significant disparity was also found between women with HZO and female controls (IRR = 3.90; 95% CI = 3.48–4.38; *p* < 0.0001; Table 2).

In the HZO group, corneal ulcer incidence rates decreased in the following order according to the comorbidities: patients wearing contact lenses (1,000/10,000 PY), with ocular allergic conditions (87.12/10,000 PY), with HIV infection (77.52/10,000 PY), with diabetes (55.13/10,000 PY), with chronic renal disease (55.12/10000 PY), and with atopy trait (45.04/10,000 PY). The IRR for corneal ulcers in HZO patients with comorbidities including diabetes (IRR = 4.77; 95% CI = 3.62–2.69; *p* < 0.0001), atopy trait (IRR = 3.83; 95% CI = 2.63–5.59; *p* < 0.0001), chronic renal disease (IRR = 5.89; 95% CI = 2.89–11.98; *p* < 0.0001), and ocular allergic conditions (IRR = 4.88; 95% CI = 2.54–9.38; *p* < 0.0001) indicate significantly greater risks than in the corresponding controls (Table 2). However, the IRR for corneal ulcers associated with HZO patients with HIV infection or contact lens wearing could not be determined, because no patients in the corresponding control groups developed corneal ulcer (Table 2).

**Figure 1:**
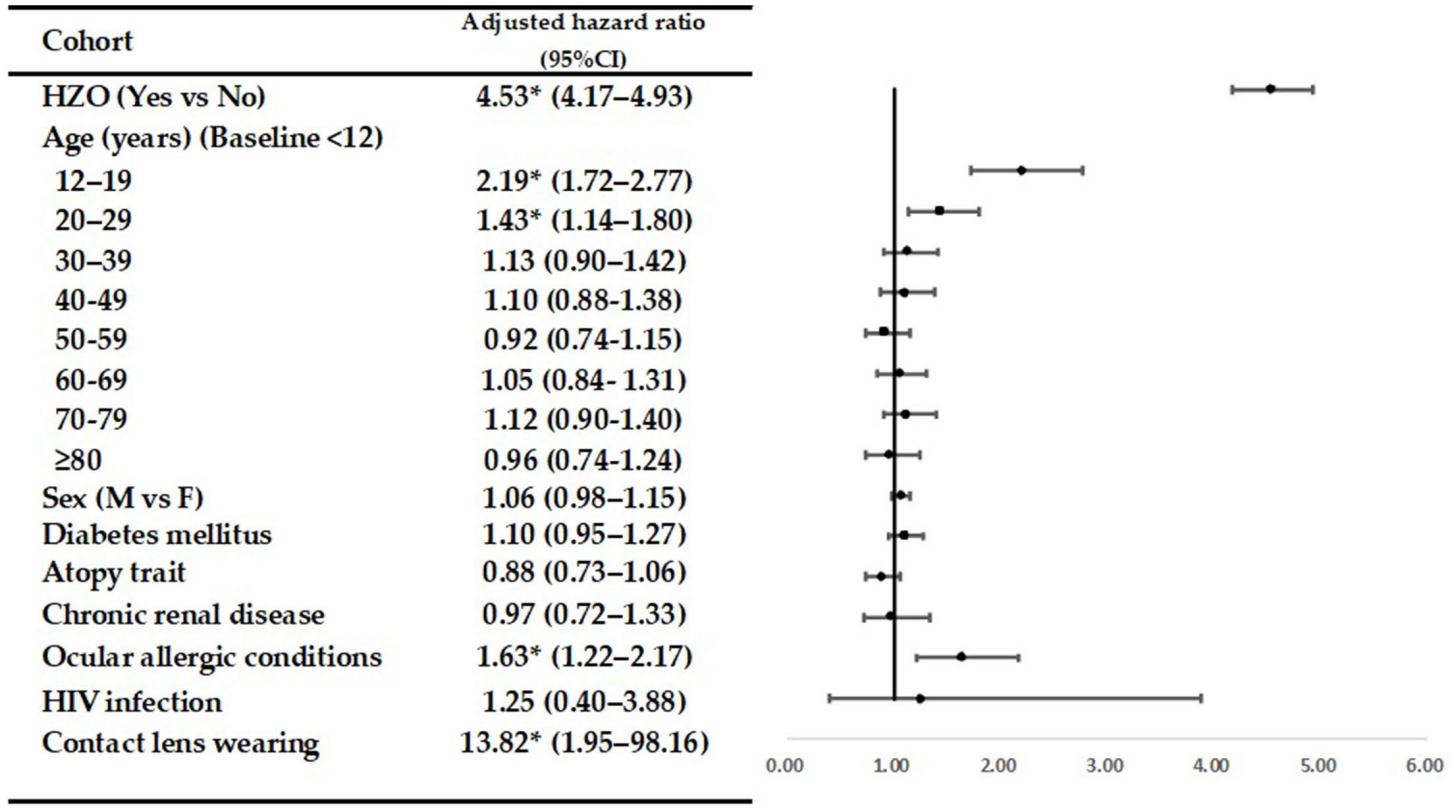
Forest plot illustrating the adjusted odds ratios.

Table 3 presents the crude and adjusted hazard ratios (HRs) for corneal ulcers during the follow-up period. The forest plot displays the adjusted odds ratios (Figure 1). Even after adjusting for age, sex, and selected comorbidities, HZO continued to be an independent risk factor for corneal ulcers (adjusted HR = 4.53; 95% CI = 4.17–4.93; *p* < 0.05). In both the HZO patients and controls, the following groups were at a higher risk of developing corneal ulcers compared to others: patients aged 12–19 (adjusted HR, 2.19; 95% CI = 1.72–2.77; *p* < 0.05); aged 20–29 (adjusted HR, 1.43; 95% CI = 1.14–1.80; *p* < 0.05); with ocular allergic conditions (adjusted HR, 1.63; 95% CI = 1.22–2.17; *p* < 0.05); and with contact lens wearing (adjusted HR, 13.82; 95% CI = 1.95–98.16; *p* < 0.05).

**Table 3:** Crude and adjusted hazard ratios with corresponding 95% confidence intervals from the Cox proportional hazards regression analyses, indicating the risk of developing a corneal ulcer in herpes zoster ophthalmicus (HZO) patients during the follow-up period in the study cohort.

Cohort	Crude hazard ratio (95% CI)	Adjusted hazard ratio (95% CI)
HZO		
Yes	4.56* (4.20–4.95)	4.53* (4.17–4.93)
No	1.00	1.00
Age (years)		
<12	1.00	1.00
12–19	2.20* (1.74–2.79)	2.19* (1.72–2.77)
20–29	1.44* (1.15–1.81)	1.43* (1.14–1.80)
30–39	1.14 (0.90–1.43)	1.13 (0.90–1.42)
40–49	1.11 (0.88–1.38)	1.10 (0.88–1.38)
50–59	0.93 (0.75–1.16)	0.92 (0.74–1.15)
60–69	1.07 (0.86–1.33)	1.05 (0.84–1.31)
70–79	1.14 (0.91–1.41)	1.12 (0.90–1.40)
≥80	0.94 (0.73–1.22)	0.96 (0.74–1.24)
Sex		
Female	1.00	1.00
Male	1.047 (0.97–1.14)	1.06 (0.98–1.15)
Comorbidity		
Diabetes mellitus	1.11 (0.97–1.27)	1.10 (0.95–1.27)
Atopy trait	1.05 (0.87–1.26)	0.88 (0.73–1.06)
Chronic renal disease	1.12 (0.83–1.52)	0.97 (0.72–1.33)
Ocular allergic conditions	2.02* (1.52–2.69)	1.63* (1.22–2.17)
HIV infection	2.55 (0.82–7.91)	1.25 (0.40–3.88)
Contact lens wearing	44.80* (6.32–317.64)	13.82* (1.95–98.16)

The adjusted hazard ratio for developing a HZO was calculated using the Cox proportional hazard regression analysis. CI, confidence interval; HIV, human immunodeficiency virus; HZO, herpes zoster ophthalmicus. ^*^ *p* < 0.05.

**Figure 2:**
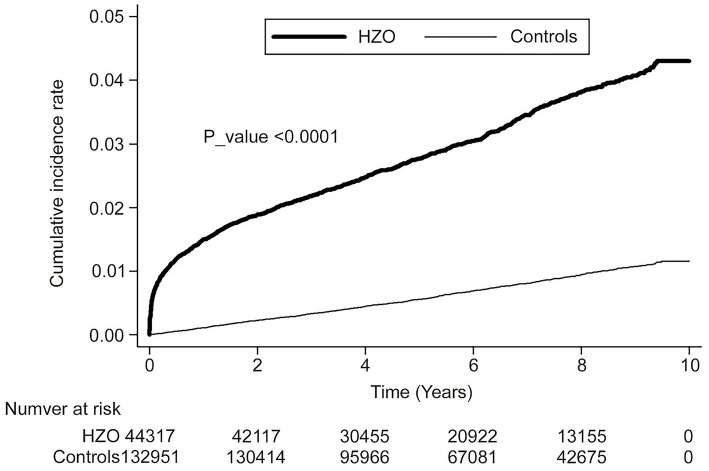
Cumulative incidence rate of corneal ulcer in patients with herpes zoster ophthalmicus and controls during the follow-up period.

Kaplan–Meier analyses revealed higher cumulative incidence rates of corneal ulcers in the HZO group than in the controls, and the log-rank test findings were also significant (*p* < 0.0001; Figure 2).

## 4 Discussion

To the best of our knowledge, this is the most extensive population-based study conducted to examine the association between HZO and the subsequent development of corneal ulcers. We analyzed data from 44,317 HZO patients and 132,951 controls matched by age, sex, and index date. Our findings revealed that the incidence rate of corneal ulcers in HZO patients was 4.59 times higher than in controls. Furthermore, the relative risk of developing a corneal ulcer among HZO patients increased by 4.53 times in the full cohort after adjusting for factors such as age, sex, diabetes, atopy trait, chronic renal diseases, ocular allergic conditions, HIV infection, and contact lens usage.

The association between HZO and corneal ulcer has been discussed in a few case reports (18, 19). Lyon and Newman (18) reported a rare complication of HZO: secondary bacterial keratitis due to the opportunistic pathogen *Branhamella catarrhalis*. That study is important for its early onset during the course of zoster and the absence of common predisposing factors, such as steroid use, contact lens wear, or prior corneal disease or surgery (18). Webb and Duke (19) reported a case involving a 61-year-old immunocompromised patient who developed severe HZO, which was complicated by a staphylococcal indolent corneal ulcer.

While the risk of secondary infection in HZO is generally recognized, it is rarely reported and is thought to primarily complicate the subacute or chronic stages of HZO (18). Our study, the largest cohort investigation to date, examined the association between HZO and corneal ulcers. We explored this relationship by analyzing the pathophysiology of common HZO complications, such as exposure keratitis and neurotrophic keratitis. Furthermore, we assessed the potential link between the frequent use of steroid eye drops in HZO patients, particularly those with stromal herpetic keratitis, and the development of secondary corneal ulcers.

Exposure keratopathy often arises from scarring and contraction of the eyelid and its margin, leading to conditions such as trichiasis, distichiasis, entropion, and lagophthalmos in patients with HZO (8). This can result in corneal dryness, further complicating HZO by exacerbating impaired corneal sensation and reduced tear production (7) These conditions can cause corneal epithelial disruption, followed by inflammation, thinning, and potential secondary infections (12). Various ocular surface disorders related to exposure keratitis, such as superficial punctate keratitis, can weaken the ocular surface barrier, heightening the risk of infectious corneal ulcers (9–11). Exposure keratitis and its associated complications may go undiagnosed in HZO patients, making it essential to assess for signs of corneal epithelial breakdown, exposure, and lagophthalmos.

Neurotrophic keratopathy, caused by a profound loss of corneal sensation, may develop months or years after the onset of HZO due to neurological damage (2). Reduced corneal sensation diminishes blinking, leading to corneal exposure and dry eye. As the cornea dries and becomes irregular, patients with pseudodendrites, keratitis, and hypoesthesia may not notice changes in vision or discomfort, allowing the condition to progress unnoticed (12). Corneal epithelial deterioration may first appear as punctate keratopathy, but if it worsens, large epithelial defects and chronic, sterile ulcers can develop, unresponsive to standard treatments. These ulcers increase the risk of secondary bacterial infections, which can cause corneal thinning and perforation (2).

Topical or systemic corticosteroids are thought to increase the risk of secondary infections, particularly in patients with HZO. Webb and Duke (19) reported a case of a patient with pemphigus vulgaris who was successfully treated with prednisone and cyclophosphamide but later developed HZO complicated by a persistent *Staphylococcus aureus* corneal ulcer, likely exacerbated by wearing a bandage soft contact lens. Furthermore, a case of secondary fungal keratitis caused by *Paecilomyces lilacinus* was reported in a patient receiving a combination of topical antibiotic and corticosteroid therapy for HZO (23). The immunosuppressive effects of corticosteroids, combined with the disruption of the ocular surface from HZO, may facilitate opportunistic infections. Topical corticosteroids, frequently prescribed to manage HZO inflammation, may impair corneal healing and increase the likelihood of superinfections, leading to corneal ulcers. The use of immunomodulatory eye drops, often indicated to control inflammation in HZO patients, can also weaken the ocular surface’s defense mechanisms, further predisposing patients to corneal ulceration (13, 14). These risks highlight the need for careful monitoring of patients receiving such treatments, especially those with preexisting corneal surface diseases or those using contact lenses.

The incidence of corneal ulcers was significantly higher in HZO patients aged 12–19 and 20–29 years (Table 2). Even after adjusting for sex and comorbidities, young HZO patients in these age groups remained at a significantly elevated risk of developing corneal ulcers (Table 3). This increased risk may be attributed to lifestyle factors common among adolescents and young adults, such as the use of contact lenses for cosmetic purposes, poor contact lens hygiene, and greater participation in outdoor activities that can lead to corneal abrasions or foreign body exposure. These factors likely contribute to the higher risk of corneal ulcers in HZO patients within this age range. In addition, contact lens wear is a well-known risk factor for corneal ulcers in younger individuals (24, 25). To evaluate the effect of this confounding factor, we considered complications associated with contact lens use, including corneal disorders due to contact lenses (ICD-9-CM code 371.82) and corneal oedema due to contact lenses (ICD-9-CM code 371.24). Our analysis revealed that the incidence of corneal ulcers was higher among patients who wore contacts (Table 2). Even after adjusting for age and comorbidities, contact lens wear remained a significant risk factor for developing corneal ulcers in both groups (Table 3).

We found that the incidence of corneal ulcers was higher in patients with ocular allergic conditions (Table 2), and these conditions remained a significant risk factor for corneal ulcer development after adjusting for age and comorbidities in both groups (Table 3). In our previous study of 171,019 patients with newly diagnosed atopic keratoconjunctivitis (AKC) compared to an equal number of matched controls, the incidence of corneal ulcers was 1.42 times higher in AKC patients (95% CI = 1.33–1.52; *p* < 0.0001). After adjusting for confounders, AKC patients were 1.26 times more likely to develop a corneal ulcer than controls (adjusted HR, 1.26; 95% CI = 1.14–1.39; *p* < 0.05) (26). We attempt to explain the association between ocular allergic conditions and corneal ulcers as follows. Ocular surface disorders in patients with ocular allergic conditions can weaken the ocular surface barrier, increasing their susceptibility to infections, including those from opportunistic pathogens found in the normal eyelid flora (27, 28). Vigorous and prolonged eye rubbing in these patients can further damage the ocular surface, raising the risk of corneal ulcers. While topical corticosteroids and immunomodulatory eye drops are effective treatments for these patients (14, 29), they also elevate the risk of developing corneal ulcers in these patients (13, 14, 30).

Several studies have shown that comorbidities such as diabetes (20), chronic renal disease (21), and HIV (22) are associated with corneal ulcers. In this cohort study, we evaluated these comorbidities in HZO patients but could not determine the association with HIV because of its low incidence. Our findings revealed that HZO patients with diabetes and those with chronic renal disease had significantly higher IRRs for corneal ulcers compared with controls (Table 2), consistent with previous studies (20, 21). This may be because of the immunocompromised state seen in diabetes and chronic renal disease, which increases susceptibility to opportunistic infections. In addition, HZO patients with an atopy trait had significantly higher IRRs for corneal ulcers than controls (Table 2). The correlation between atopy traits and corneal ulcers may be explained by ocular allergic conditions. Our previous research demonstrated a connection between atopy traits and ocular allergic conditions, and an increased risk of corneal ulcers in patients with these conditions. Consequently, patients with atopy traits are more likely to have a higher incidence rate of corneal ulcers (26).

Our study has several key strengths. First, as a population-based cohort study with a large sample size—44,317 HZO patients and 132,951 matched controls—it offers strong statistical power and precise risk assessment. Second, the likelihood of mis-diagnosis is minimized, as patients with visual problems in Taiwan typically seek care from ophthalmologists. Third, by accounting for comorbidities including diabetes, atopy traits, chronic renal disease, ocular allergic conditions, HIV, and contact lens use, and utilizing up to 10 years of longitudinal data, we significantly reduce potential confounding bias in our analysis.

This study had some limitations. First, because medical records were only available from 1996 onward, we could not verify whether the controls had a prior history of HZO before January 1996. Second, several key confounding factors, such as occupation, minor ocular trauma, previous refractive surgery, and exposure to mud or plants, were not evaluated. Although we controlled for several confounders, unmeasured lifestyle factors that may affect immune function could still have influenced the observed associations. Third, relying on ICD-9-CM codes to diagnose HZO and its associated comorbidities may have resulted in disease misclassification. Fourth, since the study was conducted in a Taiwanese population, the generalizability of these findings may be limited in other regions or among populations with different sociodemographic characteristics. A further limitation is the likely underestimation of contact lens use. Identification was based on diagnostic codes such as corneal disorder due to contact lens (ICD-9-CM 371.82) and corneal edema due to contact lens (ICD-9-CM 371.24), while most contact lens wear is not recorded in public insurance data. This may have resulted in a lower observed prevalence. Moreover, the inclusion of certain diagnostic codes for corneal ulcers, such as ICD-9-CM 370.05 (fungal keratitis) and 370.01 (marginal ulcer), may introduce some heterogeneity, as these conditions are often related to fungal or immune-mediated causes. However, these codes were retained due to the clinical relevance of HZO-related ocular surface compromise and immune suppression, which increase the risk of opportunistic and immune-related ulcers. Finally, the use of ICD-9 codes for diagnosing HZO, corneal ulcers, and other comorbidities carries the risk of potential misclassification.

In conclusion, this study demonstrated that HZO patients have a significantly higher risk of developing corneal ulcers. Even after adjusting for other confounders in the cohort, HZO remained an independent risk factor, particularly in patients aged 12–29 years, those with ocular allergic conditions, and contact lens users. These findings highlight the importance of informing HZO patients about their elevated risk of corneal ulcers.

## Data Availability

The original contributions presented in the study are included in the article/Supplementary material, further inquiries can be directed to the corresponding author.
