# Supplementary material for: Association between herpes zoster ophthalmicus and the risk of corneal ulcer
Source: Front Med (Lausanne). 2025 May 12;12:1529908. doi: 10.3389/fmed.2025.1529908 (PMC12104220; doi:10.3389/fmed.2025.1529908)
Supplement: Supplementary file 1 [file Data_Sheet_1.pdf]

Supplemental table 1: Different types of corneal ulcers.

Ulcer type	Case number (%)	Control number (%)
370.00 (corneal ulcer, unspecified)	1296 (94.10)	865 (92.22)
370.01 (marginal)	30 (2.18)	42 (4.48)
370.02 (ring)	3 (0.22)	1 (0.11)
370.03 (central)	35 (2.55)	22 (2.35)
370.04 (hypopyon ulcer)	3 (0.22)	3 (0.32)
370.05 (mycotic)	5 (0.36)	0 (0)
370.06 (perforated)	5 (0.36)	5 (0.53)

P-value=0.0212 (Fisher's exact)
